# Mortality and cancer incidence among Queensland coal mine workers: a retrospective cohort

**DOI:** 10.1136/oemed-2024-109549

**Published:** 2025-04-07

**Authors:** Deborah Catherine Glass, Stella May Gwini, Anthony Del Monaco, Lin Fritschi, Michael John Abramson, Malcolm Ross Sim, Karen Walker-Bone

**Affiliations:** 1 Monash Centre for Occupational & Environmental Health (MonCOEH), Monash University School of Public Health and Preventive Medicine, Melbourne, Victoria, Australia; 2 School of Population Health, Curtin University, Perth, Western Australia, Australia

**Keywords:** Coal Mining, Epidemiology, Mortality, Medical Oncology

## Abstract

**Objectives:**

To quantify mortality and cancer incidence among Queensland coal mine workers.

**Methods:**

A cohort of coal mine workers from Queensland was linked to Australian national death and cancer registries for the period 1983–2020. Standardised mortality (SMR) and cancer incidence (SIR) ratios were calculated for men and women compared with Australian rates. Accidental deaths, suicides and melanoma incidence were also compared with Queensland rates.

**Results:**

There were 4957 deaths among 164 622 men and 211 among 24 389 women. Overall mortality was lower than the national population for men (SMR 81 (95% CI 78 to 83)) and women (SMR 75 (95% CI 65 to 86)) and for all mine types. Mortality was significantly decreased for most death categories. Male suicide mortality was significantly increased compared with the national population, but not when compared with Queensland population rates.

There were 5492 men and 406 women with cancer. Overall cancer incidence was higher than the national population for men (SIR 107 (95% CI 104 to 109)) but not for women (SIR 99 (95% CI 90 to 108)). There were increased risks for melanoma, lip, lung, bladder and gallbladder cancers compared with the general Australian population for men and women, but the numbers of women were small. When compared with Queensland rates, the overall risk of melanoma was not increased.

**Conclusions:**

Consistent with the healthy worker effect, overall mortality was lower in this cohort. Cancer incidence was increased for men, but not women. The increased cancer risks highlight the need for further investigation.

**WHAT IS ALREADY KNOWN ON THIS TOPIC**

- Coal mine workers have a decreased all-cause mortality and decreased all cancer mortality compared with the general population.
- Cancer incidence (rather than mortality) among coal miner workers has not been well described previously.
- No previous data have been identified for mortality and cancer incidence of women coal mine workers.

**WHAT THIS STUDY ADDS**

- Overall mortality was decreased, but there was evidence that overall cancer incidence was raised for men but not for women.
- Cancers of the lip, lung, bladder and gallbladder were higher than expected for men and women, although numbers were small for women.
- Overall mortality and cancer incidence did not differ between workers at underground and open cut mines.
- Accident and suicide rates were overall higher among men but not women when compared with national rates but not when compared with Queensland state rates.

**HOW THIS STUDY MIGHT AFFECT RESEARCH, PRACTICE OR POLICY**

- The suicide risk highlights the need for improved resources for suicide prevention for men and women in the mining industry.
- Tobacco smoking is a significant risk factor for many of the cancers that were elevated in this mining cohort, independent of work exposure. Hence, emphasis on workplace antismoking campaigns could help reduce these risks.
- Sun protection policies should be continued and reinforced for coal mine workers.
- The finding of increased risk of gallbladder cancer in both men and women in the mining industry is new, but the aetiology is unknown.

## Background

 Coal mining in Queensland, Australia includes open cut and underground mines, and coal handling preparation plants (CHPPs) usually colocated at mines. In mid-2023, this industry employed 36 600 people in open cut or exploration sites and 7400 in underground mines.^1^

Workers may be exposed to coal mine dust, respirable crystalline silica and diesel engine emissions. These exposures have been associated with increased lung disease, cancer and heart disease mortality.^2^,^7^

A systematic review identified 36 studies of cancer and mortality in male coal mine workers.^8^ No cohorts of female coal mine workers were identified. The meta-analyses showed decreased all-cause mortality, standardised mortality ratio (SMR) 0.90 (95% CI 0.83 to 0.98) and decreased all-cancer mortality SMR 0.88 (95% CI 0.80 to 0.97). However, few studies identified the type of mine.

Reduced overall mortality is common in industrial cohorts, likely attributable to the ‘healthy worker effect’.^9 10^ That is, workers entering any industry are healthier than the general population, the sick are less likely to be recruited, particularly for this physically active industry.

Additionally, occupational diseases may have long latency periods between 10 and 40 years (eg, lung cancer and mesothelioma), so identification needs long follow-up periods. Some studies in the systematic review by Alif *et al*^8^ had relatively short follow-up.

A 1982 survey of Queensland coal miners identified respiratory abnormalities.^11^ Since then, pre-employment medical assessments have been mandated for all Queensland coal mine workers. From 1993, 5-yearly medical assessments were also required. Data from these assessments were submitted to and stored by the Queensland Government agency, Resources Safety and Health Queensland (RSHQ).

This study was designed to examine whether mortality and cancer incidence were higher than expected among Queensland coal mine workers.

## Methods

This is a retrospective cohort study of mortality and cancer incidence in 164 622 male and 24 389 female Queensland coal mine workers working in or after 1982. Personal data from medical assessments held by RSHQ were used to assemble the cohort. The data included given name(s) and previous name, sex, date of birth, dates of medical assessments, smoking data and mine name. Job title was recorded after 1993. Duplicates were removed and assigned sex checked; male was the default. The probability of names being female was checked against online sources, for example, https://www.popular-babynames.com/name/. If the first or second given name was judged a female one, the sex was changed to female. Where it was unclear, RSHQ was asked to check. No health data were provided by RSHQ.

Individuals whose only job title was student/cadet/intern (n=1196), visitor (n=4939) or consultant (n=890) were excluded.

The cohort start date was the 1982 survey or the participants’ first recorded assessment. Cohort members ceased to contribute person-years at death or end of the follow-up period, end of 2020 for death and 2016 for cancer. Data on the duration of employment were derived from the dates of the assessments. Data on the type of mine (open cut, underground, CHPP and unknown), mining technique (eg, long wall) and the coal type (bituminous, anthracitic) were provided by RSHQ.

We excluded 300 workers because their assessments were missing personal information, such as names (n=29) or dates of birth (n=14). The mine type was missing for 139 230 (73.7%) of participants and 210 317 (53.1%) of assessments. Other data fields were then interrogated, for example, mine name, job title and a checkbox indicating whether the individual worked underground. The mine name was recorded as ‘unknown’ or ‘various mines’ for over half (55%) of the assessments.

To identify deaths and incident cancers, the cohort was linked to the Australian Cancer Database (ACD)^12^ and the National Death Index (NDI),^13^ held by the Australian Institute of Health and Welfare (AIHW). The linkage used surnames, given names, date of birth and date of the last medical assessment. Death registration is mandatory in Australia and all deaths are recorded on the NDI. At the time of linkage, the NDI was complete from 1 January 1980 to 30Novemeber 2020 for causes of death. The ACD contains data about cancers diagnosed in Australia and was complete from 1 January 1982 to 31 December 2016. All cancers except non-melanotic skin cancers are legally required to be registered. The NDI and ACD linkages used a probabilistic linkage to identify matches with the cohort. Each possible match was given a weight indicating the probability of a true match. The possible NDI matches were returned to the research team and were independently reviewed by two researchers. This process was done by AIHW for the cancer linkage. A small number of individuals (n=5151; 3%) were excluded from the cohort because they could not be linked through the AIHW National Linkage Map, which was based on registration for Australian universal healthcare. Of these, 60% (>3000) had their first assessment in or after 2010 so would have contributed few person-years. Based on ACD data availability, cancer incidence analyses excluded workers who had their first assessment after 31 December 2016.

Australian population mortality and cancer incidence data were obtained from data hosted by the AIHW. ^14^ The cohort mortality and cancer incidence rates were compared with the national population rates to obtain age SMR and cancer incidence ratios (SIR). These ratios were calculated separately for men and women, and for specific causes of death and types of cancers. The data published by AIHW show that mortality rates for accidents, suicide and melanoma differ by geographical State. Therefore, all-cause mortality, cancer mortality, accidental death, suicide and melanoma incidence rates were also compared with Queensland-specific rates provided by the Australian Bureau of Statistics. Only underlying causes of death were analysed.

Risks were analysed separately for those under 65 or 65 years and older, in order to identify heterogeneity in the outcomes related to age and latent period of the disease. Additionally, suicide risk was analysed by era of follow-up in 5-year bands (1980–1985, 1986–1990, 1991–1995, 1996–2000, 2001–2005, 2006–2010, 2011–2015 and 2016–2020) as a way of exploring biases that could be related to timing, for example, survivor bias for the early years of follow-up, changes in exposure or diagnosis.

Sensitivity analyses examined the effects of (a) including employees recruited before 1993 with no job titles; and (b) excluding the large number of recent employees (post 2010) who could mask risks in earlier workers. Visual inspection of risk and simple Poisson regression were used to compare SMRs and SIRs across mine types. To preserve privacy, cells with fewer than six deaths or cancers are reported as <6.

## Results

The coal mine worker cohort included 189 011 workers, of whom 164 622 were men and 24 389 were women (table 1) contributing 395 861 medical assessments. The majority of workers had their first assessment after 2000 following a coal mining boom in Queensland (online supplemental figure S1). Recently, many workers have been employed via labour hire firms and/or across more than one mine, and the mine site listed on many assessments was ‘various’ or ‘unknown’. The cohort was relatively young, with an average age of less than 60 years at the end of follow-up in 2020. The average age at cohort entry was similar for men and women, at around 30 years (table 1).

**Table 1:** Description of the cohort of Queensland coal mine workers

	Men	Women
Total number of coal mine workers	164 622	24 389
Number of assessments 5 years or more apart, n (%)		
Only 1	110 860 (67.3)	20 182 (82.8)
≥2	53 762 (32.7)	4207 (17.2)
Age at first assessment, median (P_25_–P_75_) years	32.6 (25.1–42.4)	30.1 (23.4–41.6)
Period of first assessment, n (%)		
≤1985	6987 (4.2)	196 (0.8)
1986–1990	1721 (1.1)	190 (0.8)
1991–1995	3844 (2.3)	304 (1.3)
1996–2000	8278 (5.0)	542 (2.2)
2001–2005	30 626 (18.6)	3136 (12.9)
2006–2010	46 979 (28.5)	7139 (29.3)
2011–2015	48 468 (29.4)	8086 (33.2)
≥2016	17 719 (10.8)	4796 (19.7)
Death analysis summary (up to 30 November 2020)		
Person-years of follow-up	2 118 554	251 476
Number of deaths n (%)	4957 (3.0%)	211 (0.9%)
Age at death (if deceased before 30 November 2020), median (P_25_–P_75_)	57.2 (45.7–66.4)	52.6 (44.0–60.1)
Number alive at 30 November 2020	159 665	24 178
Age at 30 November 2020*, median (P_25_–P_75_) years	46.1 (36.8–56.5)	41.3 (33.5–52.0)
Cancer incidence analysis summary (up to 31 December 2016)†		
Number of coal mine workers included in analysis	149 607	20 340
Person-years of follow-up‡	1 514 169	161 447
Number of workers with a cancer diagnosis (%)	5492 (3.7%)	406 (2.0%)
Number of people with, n (%)		
1 primary cancer	5069 (92.3)	385 (94.8)
2 primary cancers	400 (7.3)	21 (5.2)
3+ primary cancers	23 (0.44)	0
Age at diagnosis of first cancer§, median (P_25_–P_75_) years	56.5 (48.3–63.3)	47.2 (39.1–54.4)
Smoking data (most recent known status)		
Never smoked	75 102 (45.6%)	13 068 (53.6%)
Current smoker	45 933 (27.9%)	6311 (25.9%)
Ex-smoker	42 451 (25.8%)	4804 (19.7%)
Not recorded	1136 (0.7%)	206 (0.8%)
Site type (more than one site type possible)		
Open cut	69 224	12 094
Underground	34 079	2983
CHPP	2931	859
Unknown for all assessments	68 389	9512

* Includes only those alive at end of follow-up.

† Excludes those whose first assessment was after 2016.

‡ This includes time from first assessment to the earliest of date of death or 31 December 2016 for all workers.

§ Excludes cancers diagnosed prior to first assessment.

CHPP, Coal Handling Preparation Plants.

The study included participants from 34 underground mines, 66 open cut and 2 sites with both. Four were exploration sites and 34 sites had CHPPs. Of the underground mines, 11 were longwall mines, the remainder were ‘bord and pillar’ or unidentified. The coal mined at these sites was almost all bituminous. Only five of the mines had some semianthracitic deposits.

Table 1 shows that about one-third of workers (35.7% of men and 35.8% of women) had only worked in an open cut mine, while 20.2% of men and 10.1% of women had ever worked in an underground mine. A small number of workers (1.8% of men and 3.2% of women) had ever held a CHPP job. For the remaining 41.2% men and 33.2% women, the mine type could not be classified for any of their assessments.

### Mortality

There were 4957 (3.0%) deaths among men and 211 (0.9%) deaths among women. There was significantly reduced overall mortality for men and women compared with the national population. For men, the SMR was 81, 95% CI 78 to 83 and for women 75, 95% CI 65 to 86 (table 1). Compared with Queensland rates, the SMRs for men for all causes of death and all malignancies were similar (SMR_all-cause_ 78, 95% CI 76 to 80; SMR_all-malignancies_ 86, 95% CI 83 to 90). The overall mortality findings compared with national rates were robust to sensitivity analyses that excluded those with first assessment dates before 1993 (SMR 82, 95% CI 79 to 84 for men, SMR 75, 95% CI 66 to 86 for women) or those with first recorded assessment dates post 2009 (SMR 82, 95% CI 80 to 85 for men, SMR 81, 95% CI 69 to 95 for women).

There were reduced SMRs for most major causes of mortality (table 2), for men and women. There were few deaths from non-malignant respiratory disease. Deaths from lung diseases due to dust (International Classification of Diseases (ICD)-10 code J60–J67) (n=6 in total) were significantly increased in men aged under 65 years (SMR 394, 95% CI 127 to 1222) but not in the older group (SMR 97, 95% CI 31 to 300) (online supplemental table S1).

**Table 2:** Comparisons of mortality rates in male and female coal mine workers with the Australian population rates

Cause of death categories*(ICD-10 Code)	MenN=164 622PY=2 118 553			WomenN=24 389PY=251 476		
	O†	E	SMR (95% CI)	O†	E	SMR (95% CI)
All causes of death (A00–Z99)	4957	6143	81 (78 to 83)	211	282	75 (65 to 86)
All Malignancies (C00–C97, D45–D46, D47.1, D47.3, D47.4, D47.5)	1888	2093	90 (86 to 94)	99	127	78 (64 to 95)
All metabolic (E00–E99)	123	228	54 (45 to 64)	6	10	57 (26 to 128)
Diabetes (E10–E14)	89	154	58 (47 to 71)	<6		81 (34 to 194)
All mental and behavioural (F00–F99)	37	100	37 (27 to 51)	<6		32 (5 to 228)
Dementia (F01, F03)	19	36	52 (33 to 82)	0		
All nervous system (G00–G99)	93	209	44 (36 to 55)	6	11	53 (24 to 118)
Alzheimer disease (G30)	12	18	67 (38 to 118)	0		
Parkinson disease (G20–G22)	11	22	50 (28 to 90)	0		
All circulatory (I00–I99)	944	1306	72 (68 to 77)	30	37	82 (57 to 117)
IHD (I20–I25)	597	777	77 (71 to 83)	11	13	86 (48 to 155)
Cerebrovascular (I60–I69)	113	185	61 (51 to 73)	7	10	72 (35 to 152)
Other heart disease (I05–I09, I11, I13, I26, I27, I30–I52)	172	263	65 (56 to 76)	9	11	83 (43 to 160)
All respiratory (J00–J99)	221	304	73 (64 to 83)	10	14	70 (37 to 129)
COPD (J40–J44)	137	161	85 (72 to 101)	<6		28 (7 to 112)
Asthma (J45, J46)	8	18	45 (23 to 90)	<6		236 (89 to 629)
Lung diseases due to dust (J60–J67)	6	4	155 (70 to 346)	0		
All digestive (K00–K93)	136	291	47 (40 to 55)	8	12	66 (33 to 131)
Liver disease (K70–K77)	87	207	42 (34 to 52)	8	8	95 (48 to 190)
All urinary (N00–N99)	26	53	50 (34 to 73)	<6		78 (20 to 312)
Kidney failure (N17–N19)	19	34	56 (36 to 88)	<6		69 (10 to 489)
All injury and trauma (V01–Y98)	1336	1261	106 (100 to 112)	45	47	96 (72 to 128)
Accidents (V01–X59, Y85–Y86)	674	643	105 (97 to 113)	22	23	94 (62 to 143)
Suicide (X60–X84)	614	520	118 (109 to 128)	18	19	96 (60 to 152)
All other causes	144	299	48 (41 to 57)	<6		18 (7 to 47)

* Specific cause of death for nine male workers was unknown.

† 0 means no deaths.

COPD, Chronic Obstructive Pulmonary Disease; ICD, International Classification of Diseases; IHD, Ischaemic Heart Disease; O, observed number of deaths; PY, person-year; SMR, standardised mortality ratio.

Deaths from external causes, accidents and suicides were increased for men but not for women (table 2). Mortality from external causes was significantly higher among men under 65 years compared with the Australian population but not for those over 65 (online supplemental table S1).

The accidental death rate (ICD190 codes V01–X59, Y85–Y86) for men (SMR 105, 95% CI 97 to 113) was lower compared with Queensland rates (SMR_Queensland_ 96, 95% CI 89 to 104) (online supplemental figure S2). Most (59%) of the accidental deaths were transport accidents and 21% accidental poisonings. The remainder of deaths included slips, trips and falls, electrocutions and crushing etc.

**Figure 1:**
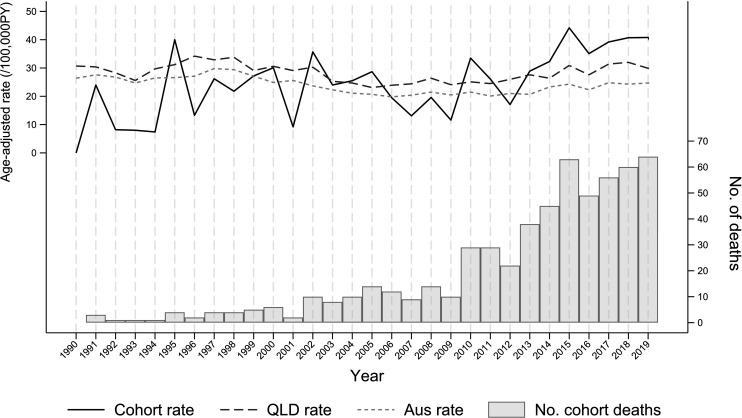
Age-standardised suicide rates for male coal miners compared with Queensland and Australian population from 1990 to 2020 (comparison data provided by ABS) (Trend: Coal miners β=0.76/100 000 /year, p<0.001; Australian population β=−0.18/100 000 /year, p=0.001; Queensland population β=−0.07/100 000 /year, p=0.257. ABS, Australian Bureau of Statistics; QLD, Queensland.

Suicide was significantly increased among men under 65 years compared with the Australian population but not for those over 65. The year-by-year changes in the cohort suicide rates are shown in figure 1 together with the Australian and Queensland rates. Analyses by 5-year bands showed that the suicide rate for men was reduced or similar to the Queensland rates between 1980 and 2010. Between 2011 and 2015, the SMR_Queensland_ was 103, 95% CI 90 to 119 (n=197) and between 2016 and 2020 SMR_Queensland_, it was 108, 95% CI 96 to 122 (n=264).

Analysis of overall mortality by mine type (table 3) showed that none of the death categories were in excess compared with the Australian population. However, SMRs were higher for male workers where the mine type was unknown and lower for women working at open cut and underground sites.

**Table 3:** All-cause mortality and all-cancer incident rates for male and female workers employed after 1992*, by Mine Type

By mine type	Male workers			Female workers		
**All-cause mortality**	**N (PY)**	**O**	**SMR (95% CI)**	**N (PY)**	**O**	**SMR (95% CI)**
Only worked in open cut (excludes CHPP and underground)	58 799 (779 630)	1985	79 (76 to 83)	10 908 (112 434)	100	80 (66 to 98)
Ever underground (excludes CHPP)	33 284 (440 467)	756	69 (64 to 74)	2917 (30 420)	8	27 (14 to 55)
Ever CHPP	2929 (47 217)	78	52 (42 to 65)	659 (7460)	6	64 (29 to 143)
Only unknown	67 875 (705 632)	1810	94 (90 to 99)	9448 (87 970)	88	84 (68 to 103)
**Incidence of all malignancies**	**N (PY)**	**O**	**SIR (95% CI)**	**N (PY)**	**O**	**SIR (95% CI)**
Only worked in open cut (excludes CHPP and underground)	52 521 (566 684)	2655	111 (107 to 115)	8732 (73 597	185	95 (82 to 110)
Ever underground (excludes CHPP)	30 858 (317 168)	1056	107 (101 to 114)	2457 (19 885)	44	96 (71 to 129)
Ever CHPP	2784 (36 264)	125	87 (73 to 104)	787 (6741)	10	66 (35 to 122)
Only unknown	61 676 (454 039)	1731	104 (100 to 109)	8104 (53 174)	157	103 (88 to 120)

* SMRs and SIRs are standardised for age (in 5-year age bands).

CHPP, coal handling preparation plant; O, observed number of deaths or cancers; PY, person-year; SIR, standardised incidence ratio; SMR, standardised mortality ratio.

### Cancer incidence

Workers whose first assessment was after 2016 were not included in the cancer incidence analyses because the ACD was complete only until 2016. This excluded 10% of men (n=15 017) and 16.6% of women (n=4049). Among the remaining 149 607 men and 20 340 women, there were 5940 cancers identified in men and 427 cancers in women, with 444 workers diagnosed with two or more primary cancers. Overall cancer incidence was increased among men but not women (table 4).

**Table 4:** Comparisons of cancer incidence rates in male and female coal mine workers with the Australian population rates

Cancer categories(ICD10 code)	All menN=149 607; PY=1 514 169			All womenN=20 339; PY=161 447		
	O	E	SIR (95% CI)	O*	E	SIR (95% CI)
All malignancies (C00–C97, D45–D46, D47.1, D47.3–D47.5)	5940	5568	107 (104 to 109)	427	433	99 (90 to 108)
Lip, oral cavity and Pharynx (C00–C14)	329	297	111 (99 to 123)	8	8	98 (49 to 196)
Lip (C00)	125	90	138 (116 to 165)	<6		178 (57 to 552)
Pharynx (C09–C14)	100	93	107 (88 to 131)	<6		116 (29 to 463)
Digestive organs (C15–C25)	1046	1079	97 (91 to 103)	51	51	101 (77 to 133)
Oesophagus (C15)	71	74	96 (76 to 121)	0		
Stomach (C16)	80	104	77 (62 to 96)	<6		104 (39 to 277)
Colorectal (C18–C21)	661	631	105 (97 to 113)	36	35	102 (74 to 142)
Liver (C22)	83	114	73 (59 to 91)	0		
Gallbladder (C23–C24)	30	23	130 (91 to 186)	<6		282 (106 to 752)
Pancreas (C25)	89	104	86 (70 to 106)	<6		102 (42 to 245)
Respiratory and intrathoracic organs (C30–C38)	491	463	106 (97 to 115)	23	20	114 (76 to 171)
Larynx (C32)	56	46	122 (94 to 158)	0		
Lung (C33–C34)	416	397	105 (95 to 115)	22	19	117 (77 to 178)
Melanoma (C43)	987	752	131 (123 to 140)	76	57	134 (107 to 168)
Mesothelioma (C45)	36	28	131 (94 to 181)	0		
Breast (C50)	9	10	91 (47 to 174)	150	156	96 (82 to 113)
Female reproductive organs (C51–C58)	NA			34	48	71 (50 to 99)
Male reproductive organs (C60–C63)	1729	1594	108 (103 to 114)	NA		
Prostate (C61)	1577	1422	111 (106 to 117)	NA		
Testis (C62)	144	161	89 (76 to 105)	NA		
Urinary tract (C64–C68)	320	314	102 (91 to 114)	9	10	92 (48 to 177)
Kidney (C64)	201	205	98 (85 to 113)	<6		38 (12 to 118)
Bladder (C67)	103	95	108 (89 to 131)	<6		330 (137 to 792)
Brain and other CNS (C70–C72)	126	115	109 (92 to 130)	<6		45 (15 to 141)
Thyroid and other endocrine glands (C73–C75)	99	97	102 (83 to 124)	30	31	98 (69 to 140)
Lymphoid, haematopoietic and related tissue (C81–C96, D46)	490	545	90 (82 to 98)	31	31	101 (71 to 143)
Non-Hodgkin's lymphoma (C82–C86)	201	239	84 (73 to 97)	13	13	101 (58 to 173)
Multiple myeloma (C90)	72	70	102 (81 to 129)	<6		125 (47 to 332)
Leukaemia (C91–C95)	182	171	106 (92 to 123)	6	9	69 (31 to 154)
Lymphoid leukaemia (C91)	120	94	127 (106 to 152)	<6		56 (14 to 223)
Myeloid leukaemia (C92)	47	63	75 (56 to 100)	<6		91 (34 to 243)
Other cancers (C26, C39, C40-41, C44, C46, C47–C49, C69, C76, D45, D47–D49)	195	202	96 (84 to 111)	7	12	60 (28 to 125)

* 0 means no instances.

ICD, International Classification of Diseases; NA, not available; O, observed number of deaths or cancers; PY, person-year; SIR, standardised incidence ratio.

Cancers of the lip, lung, bladder and gallbladder were increased in both men and women, although for women the numbers were small (table 4). In addition, prostate and laryngeal cancer, mesothelioma and lymphoid leukaemia were increased in men. Numbers were too small for robust analyses for many cancer types in women. Melanoma risks were increased when compared with the national rates, but not when compared with Queensland rates for either men (SIR_Queensland_ 89, 95% CI 84 to 95) or women (SIR_Queensland_ 88, 95% CI 70 to 110).

Analysis of cancer incidence by mine type (table 3) shows an increase for men at all mine types except CHPP and for women at unknown mine types, relative to the national population.

The overall cancer incidence rates for all male workers under 65 years (SIR 106, 95% CI 103 to 110) and over 65 years (SIR 108, 95% CI 102 to 114) were higher than the Australian population (online supplemental table S2). Increased risks were observed for lip cancer, melanoma and prostate cancer among men under 65 years, but not over 65 years. Conversely, lung cancer, mesothelioma, leukaemia and lymphoid were significantly increased in male workers after the age of 65 years but not in the younger age group. Rates of laryngeal cancer were increased in younger and older men.

## Discussion

There was reduced overall mortality in this coal mine worker cohort, likely attributable to the ‘healthy worker effect’,^9^ seen in other occupational cohorts.^15^ The Australian Health Watch cohort of petroleum industry workers had a similar overall SMR for men of 72 (95% CI 69 to 75).^16^ No difference was seen between the <65 and 65+ SIRs but the short follow-up for many workers means that this bias could not be ruled out.

The overall male SMR was higher for men over 65 years than for younger men, although the risk of suicide was higher in the younger men. Compared with the Queensland population, there was no increased risk of suicide. AIHW data show that suicide rates were higher in Queensland than in the rest of Australia.^17^ This may be partially explained by the higher rate of suicides in regional/remote areas.^18^ Our data suggested an increase in suicide in recent years. Queensland coronial data showed that suicide rates were higher in the mining industry than among other employed people^19 20^ and that rates have been increasing since 2011.^20^ However, a 2015 study did not identify increased suicide among Queensland miners.^21^

The accidental death rate for male coal mine workers was higher than the Australian population but similar to that for men in Queensland. A recent Safe Work Australia report identified 10 fatalities in the whole mining sector in Queensland between 2017 and 2021,^22^ and 10 between 2013 and 2017.^23^ 59% of the accidental deaths were transport accidents which may occur when commuting to or from work or during leisure time. Driving distances are often higher in rural areas so that there is more opportunity for road accidents.

Overall cancer incidence was increased in men compared with the Australian population. A previous cohort study of 23 630 male coal miners from New South Wales Australia included workers recruited in or after 1973 and followed until 1991.^24^ It found an overall cancer SIR of 82 (95% CI 73 to 92),^24^ lower than the male SIR in this study. The highest SIRs in the New South Wales (NSW) cohort were for melanoma (113, 95% CI 90 to 139), cancers of the lip (102, 95% CI 49 to 187), larynx (102, 95% CI 37 to 221) and colon (100, 95% CI 66 to 145). These cancers were also increased in this study. Cancers of the lung and stomach were not significantly increased in the NSW study,^24^ the meta-analysis^8^ or this study. Lung, lip, bladder^25^ and gallbladder^26^ cancer incidence and perhaps melanoma mortality^27^ have been shown to increase with tobacco smoking.

Higher rates of smoking-related deaths and some cancers may result from higher rates of smoking. 28% of men and 26% of women were smokers at their most recent assessment. Australian smoking rates have declined steadily from 1980 when about 40% of men and 30% of women over 18 were regular smokers. Approximately 50% of the cohort had their first assessment in or after 2006 so appropriate national comparison smoking rates would be from 2007 when about 22% of men and 18% of women were smokers.^28^

Rates of smoking have been about 3% higher in Queensland than in the rest of Australia for some years,^29^ more in rural/regional compared with urban areas. In 2022, the Australian rural/regional smoking rates were as high as 20%, compared with 7% in cities.^29^

Cholangiocarcinoma (the predominant type of gallbladder cancer) has been associated with exposure to 1,2-dichloropropane, asbestos, endocrine-disrupting compounds and rotating shift work.^30^ Some coal mine workers could have exposure to these agents, and the majority of mining machines run continuously for weeks/months, thus relying on shift work. Additionally, increased risk of bladder cancer has been associated with exposure to polycyclic aromatic hydrocarbons and diesel exhaust as well as tobacco smoking and perhaps decreased fluid intake.^31 32^

Melanoma rates were higher than expected for men and women compared with national rates, but not when compared with Queensland rates. This suggests that the risks are related to state of residence. Indeed, early life exposures are an important risk factor for melanoma.^33^ Much open cut mining and CHPP work is done outside, highlighting the need for UV solar protection measures, including the use of enclosed cabs.

Cancer risks were similar for open cut and underground mines, raised for men but not women. The risks were lower for CHPP workers. Almost two-thirds (63.3%) of workers at unknown mine types either only had one assessment (or had all assessments within 5 years), that is, appeared to be short-term or recent employees. Men and women whose jobs were only at unknown site type had higher overall mortality than other groups. Short-term employees have been observed to have higher mortality than longer-term employees.^34^

There were 24 389 women in the cohort with 253 567 person-years of follow-up. This is much larger than most other coal mine worker cohorts where few, if any, women were included. Only a minority of these women had worked for more than 5 years in the industry; few had more than one assessment. However, with increasing participation of women in the industry, future studies will have more power to investigate risks.

A strength was a near-complete enumeration of all Queensland coal mine workers employed after 1982, minimising the risk of ascertainment bias. There were only 300 people who had assessments with missing information that could not be included in the cohort. These were mainly assessments from after 2016 and as these were recent employees, their exclusion was unlikely to have significantly affected the findings.

The NDI and ACD are an advantage in this study. Cancer and death registration is mandatory across Australia and is virtually complete.^35^ Matching the cohort to the NDI and ACD was a probabilistic process, but the availability of middle names and dates of last known assessment improved the probability of a correct match. Previous validation studies of the NDI have found good sensitivity and specificity for NDI linkages, with sensitivity between 88%^36^ and 95%^37^ and specificity of about 98%.^36 37^

Several cancers now respond to treatment, so incidence is a better measure of disease than mortality. In this study, there were 5950 men and 427 women with cancer but only 1888 men and 99 women who had died from cancer. Most previous cohort studies of coal mine workers only reported cancer mortality rather than incidence. The meta-analysis suggested that the risk of overall cancer mortality was approximately 10% lower than expected.^8^ No cancer categories showed statistically significant increases in the meta-analysis. Stomach cancer mortality showed an 11% increased risk in coal mine workers compared with expected values. In this study, stomach cancer incidence was not raised for men or women overall, although men aged over 65 had 10% higher risk than the Australian population of matched age, while risk among those under 65 was 33% lower.

The limitations of the study include the youth of the cohort and the relatively short follow-up period for many workers. More than 65% had their first assessment in or after 2006. Many diseases have a long latent period,^38 39^ so it is probably too early for work-related cancers to have been diagnosed, particularly as cancers were only matched to 2016. Excess lung cancers and mesotheliomas were seen in the over 65s but not in the younger group. However, the sensitivity analyses excluding those with first assessment dates post 2010 suggest that the large number of recent employees have not skewed the results.

The exact start and end dates of employment as a coal mine worker in Queensland were not available but were derived from the first and last assessment dates. As a result, cancers occurring before the first assessment date, but after the start of employment could not be included, potentially reducing the power of the study. Employment in other industries (including other types of mine) was not captured, nor was employment as a coal miner in other states or overseas. A previous study showed that almost 22% of Queensland coal mine workers with dust lung disease had also worked in other types of mines, and more than half had worked in mines outside Queensland.^40^

## Conclusions

Compared with the general Australian population, the mortality in these largely bituminous coal mine workers cohort was significantly reduced for both men and women. Deaths from external causes were increased for men, but not women when compared with national rates, but the increases were no longer evident when compared with Queensland rates.

Overall cancer incidence was increased for men but not women. Increased cancer risks were seen in both men and women for lung, lip, bladder, gallbladder cancers and melanoma.

Neither all-cause mortality nor cancer incidence differed greatly between open cut and underground mine types but were decreased compared with the Australian population for men and women in the CHPP plants.

As the cohort ages, more cancers and deaths will accumulate, and this will increase the precision of the risk estimates. Future linkages are likely to give more robust estimates of mortality risk, particularly for women.

## Supplementary material

10.1136/oemed-2024-109549 online supplemental file 1

## Data Availability

No data are available.
