# Supplementary material for: Mortality and cancer incidence among Queensland coal mine workers: a retrospective cohort
Source: Occup Environ Med. 2025 Apr 7;82(3):e109549. doi: 10.1136/oemed-2024-109549 (PMC12171497; doi:10.1136/oemed-2024-109549)
Supplement: online supplemental file 1 [file oemed-82-3-s001.pdf]

Supplementary Figures

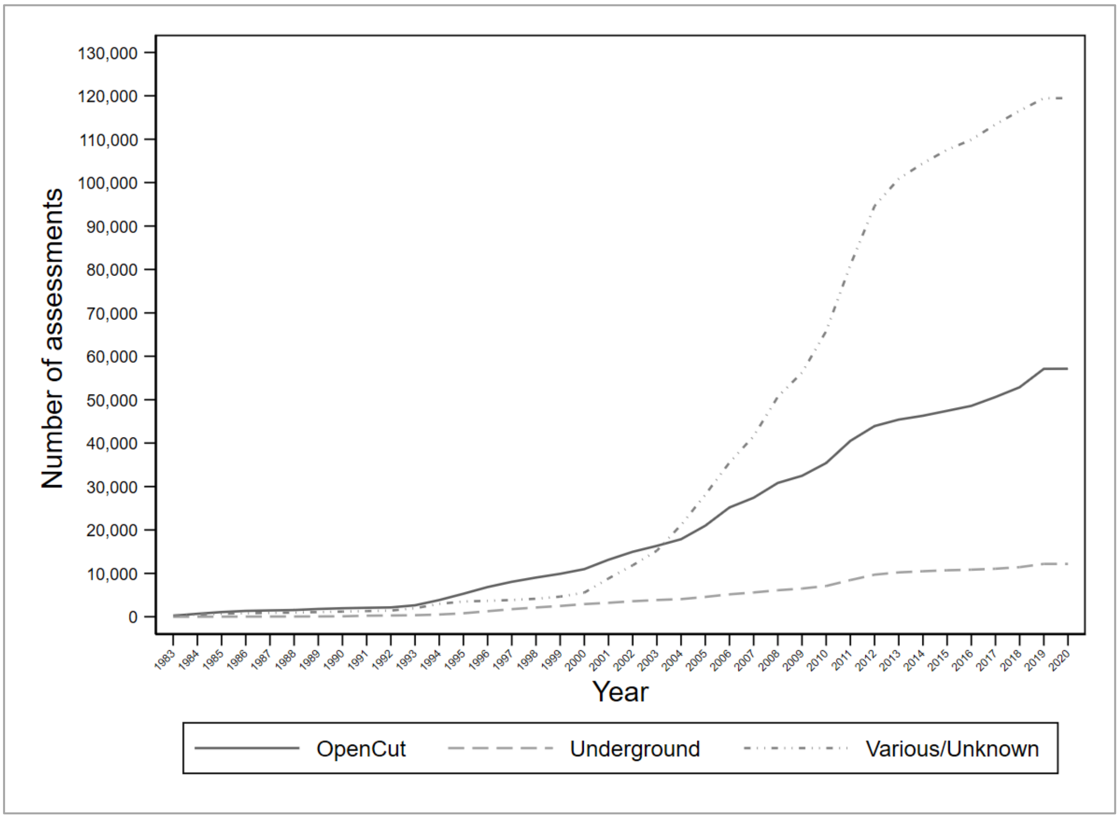

Figure S1: Cumulative number of first assessments by Mine Type and year

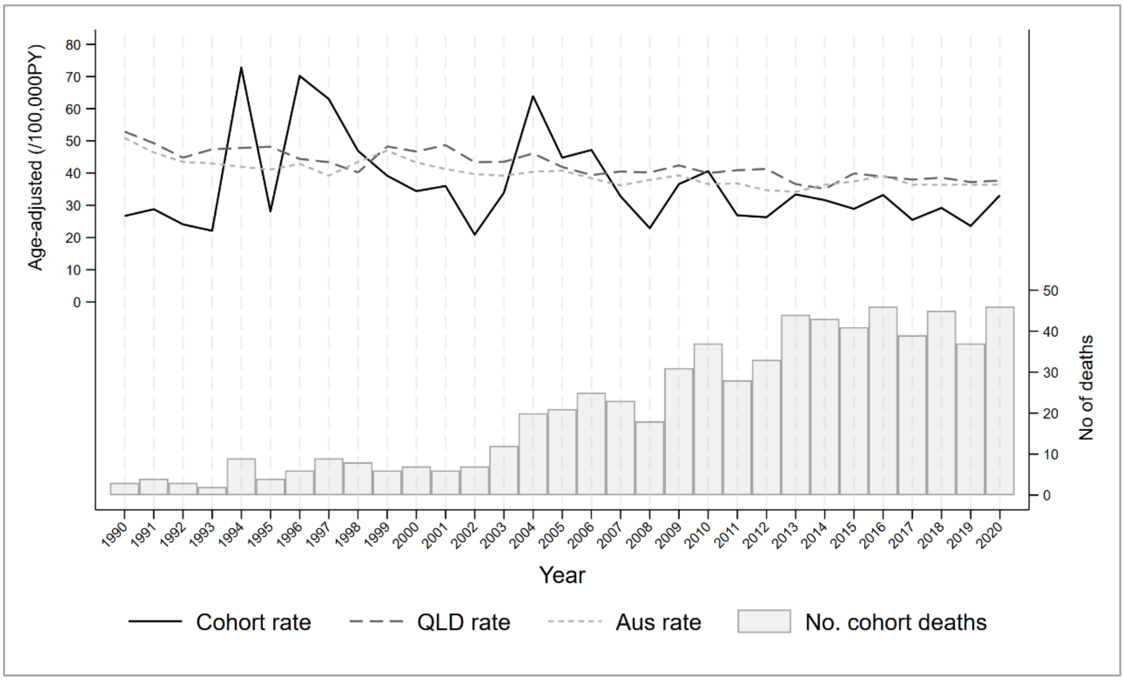

Figure S2: Age-standardised accident rates for male coal miners compared to Queensland and Australian population from 1990-2020

[TREND: Coal miners β= -0.44/100,000/year, p=0.125; Australian population β= -0.35/100,000/year, p<0.001; Queensland population β= -0.41/100,000/year, p<0.001]

Supplementary Table

Table S1: Comparisons of mortality rates in male coal mine workers with the Australian population rates, by age group

Cause of death categories	< 65 N=164,067; PY=2,021,570			65+ N=17,255; PY=96,983		
	O	E	SMR (95% CI)	O	E	SMR (95% CI)
**All causes of death**	3539	4513	78 (76 - 81)	1418	1631	87 (83 - 92)
**All Malignancies**	1155	1386	83 (79 - 88)	733	707	104 (96 - 111)
**All Metabolic**	83	154	54 (44 - 67)	40	74	54 (39 - 73)
Diabetes	57	97	58 (45 - 76)	32	57	56 (40 - 80)
**All Mental and Behavioural**	18	62	29 (18 - 46)	19	38	51 (32 - 79)
**All Nervous System**	51	135	38 (29 - 50)	42	74	57 (42 - 77)
**All Circulatory**	656	915	72 (66 - 77)	288	391	74 (66 - 83)
IHD	437	562	78 (71 - 85)	160	215	74 (64 - 87)
Cerebrovascular	66	115	57 (45 - 73)	47	70	67 (50 - 89)
Other Heart Disease	114	183	62 (52 - 75)	58	79	73 (56 - 94)
**All Respiratory**	78	162	48 (39 - 60)	143	142	101 (86 - 119)
COPD	41	75	55 (40 - 75)	96	86	111 (91 - 136)
Asthma	7	16	44 (21 - 93)	<6		50 (7 - 356)
Lung Disease Due to Dust	<6		394 (127 - 1222)	<6		97 (31 - 300)
**All Digestive**	99	232	43 (35 - 52)	37	59	63 (46 - 87)
Liver Disease	69	178	39 (31 - 49)	18	30	61 (38 - 97)
**All Urinary**	6	28	21 (9 - 47)	20	24	83 (54 - 129)
**All Injury and Trauma**	1283	1200	107 (101 - 113)	53	61	86 (66 - 113)
Accidents	638	604	106 (98 - 114)	36	40	91 (65 - 126)
Suicide	597	503	119 (110 - 129)	17	17	99 (62 - 160)
**All Other Causes**	102	238	43 (35 - 52)	42	61	69 (51 - 93)

Abbreviations: N = number of workers; O = observed number of deaths s; PY = person-years; SMR = standardized mortality ratio

Table S2: Comparisons of cancer incidence rates in male coal mine workers with the Australian population rates, by age group

Cancer Categories	<65 years *N= 149,132; PY= 1,466,127*			>65 years *N= 10,157; PY= 48,042*		
	O	E	SIR (95% CI)	O	E	SIR (95% CI)
**All Malignancies**	4700	4418	106 (103 - 110)	1240	1150	108 (102 - 114)
**Lip, Oral Cavity and Pharynx**	287	264	109 (97 - 122)	42	33	126 (93 - 171)
Lip	120	82	146 (122 - 174)	<6		62 (26 - 150)
Pharynx	85	83	102 (83 - 127)	15	10	150 (90 - 249)
**Digestive Organs**	806	846	95 (89 - 102)	240	232	103 (91 - 117)
Oesophagus	62	57	108 (85 - 139)	9	17	53 (27 - 101)
Stomach	55	82	67 (52 - 88)	25	23	110 (74 - 163)
Colorectal	511	494	103 (95 - 113)	150	137	110 (94 - 129)
Colon	277	264	105 (93 - 118)	93	84	110 (90 - 135)
Rectum	229	215	106 (94 - 121)	55	50	111 (85 - 144)
Liver	69	95	73 (58 - 92)	14	19	74 (44 - 125)
Gallbladder	20	16	122 (79 - 189)	10	7	151 (81 - 280)
Pancreas	64	78	82 (64 - 104)	25	25	98 (66 - 145)
**Respiratory and Intrathoracic Organs**	333	335	99 (89 - 111)	158	128	123 (105 - 144)
Larynx	45	37	122 (91 - 164)	11	9	119 (66 - 215)
Lung	272	280	97 (86 - 109)	144	117	123 (105 - 144)
**Melanoma**	893	649	138 (129 - 147)	94	103	91 (75 - 112)
**Mesothelioma**	18	17	106 (67 - 169)	18	11	170 (107 - 270)
**Breast**	6	8	78 (35 - 173)	<6		137 (44 - 426)
**Male Reproductive Organs**	1297	1187	109 (103 - 115)	432	407	106 (97 - 117)
Prostate	1146	1017	113 (106 - 119)	431	405	107 (97 - 117)
Testis	143	161	89 (76 - 105)	<6		134 (19 - 954)
**Urinary Tract**	255	249	103 (91 - 116)	65	65	99 (78 - 127)
Kidney	174	174	100 (86 – 116)	27	30	89 (61 - 129)
Bladder	74	65	114 (91 - 143)	29	30	96 (67 - 138)
**Brain and Other CNS**	115	103	112 (93 - 134)	11	12	89 (49 - 160)
Brain	113	99	115 (95 - 138)	11	12	91 (51 - 165)
**Thyroid and Other Endocrine**	90	90	100 (81 - 123)	9	7	123 (64 - 236)
Thyroid	85	84	102 (82 - 126)	9	7	132 (69 - 253)
**Unknown Site**	64	63	102 (80 - 130)	19	18	103 (66 - 162)
**Lymphoid, Haematopoietic & Related Tissue**	383	453	85 (76 - 93)	107	92	116 (96 - 141)
Hodgkin	27	46	59 (41 - 86)	<6		140 (45 - 434)
Non-Hodgkin Lymphoma	161	200	80 (69 - 94)	40	39	104 (76 - 141)
Diffuse Non-Hodgkin	78	96	81 (65 - 101)	16	21	77 (47 - 125)
Multiple Myeloma	53	54	98 (75 - 129)	19	17	115 (73 - 180)
Leukaemia	138	139	99 (84 - 117)	44	32	139 (103 - 187)
Lymphoid Leukaemia	92	75	122 (99 - 148)	28	19	148 (102 - 214)
Myeloid Leukaemia	38	54	70 (51 - 96)	9	8	108 (56 - 207)
**Other Cancers**	151	162	93 (79 - 109)	42	40	105 (78 - 142)
Myelodysplastic Syndrome	14	15	92 (55 - 156)	8	12	69 (35 - 139)
Connective tissue	36	40	90 (65 - 125)	<6		76 (28 - 202)

Abbreviations: N = number of workers; O = observed number of cancers; PY = person-years; SIR = standardized incidence ratio
